# Age-Specific Sex-Related Differences in Infections: A Statistical Analysis of National Surveillance Data in Japan

**DOI:** 10.1371/journal.pone.0042261

**Published:** 2012-07-27

**Authors:** Nobuoki Eshima, Osamu Tokumaru, Shohei Hara, Kira Bacal, Seigo Korematsu, Shigeru Karukaya, Kiyo Uruma, Nobuhiko Okabe, Toyojiro Matsuishi

**Affiliations:** 1 Department of Biostatistics, Faculty of Medicine, Oita University, Yufu, Oita, Japan; 2 Department of Neurophysiology, Faculty of Medicine, Oita University, Yufu, Oita, Japan; 3 Editorial Bureau, The Yomiuri Shimbun Osaka (newspaper), Osaka, Japan; 4 Medical Programme Directorate, Faculty of Medical and Health Sciences, University of Auckland, Auckland, New Zealand; 5 Department of Pediatrics and Child Neurology, Faculty of Medicine, Oita University, Yufu, Oita, Japan; 6 Department of Pediatrics and Child Health, Kurume University School of Medicine, Kurume, Fukuoka, Japan; 7 Infectious Disease Surveillance Center, National Institute of Infectious Diseases, Shinjuku, Tokyo, Japan; Chancellor College, University of Malawi, Malawi

## Abstract

**Background:**

To prevent and control infectious diseases, it is important to understand how sex and age influence morbidity rates, but consistent clear descriptions of differences in the reported incidence of infectious diseases in terms of sex and age are sparse.

**Methods and Findings:**

Data from the Japanese surveillance system for infectious diseases from 2000 to 2009 were used in the analysis of seven viral and four bacterial infectious diseases with relatively large impact on the Japanese community. The male-to-female morbidity (MFM) ratios in different age groups were estimated to compare incidence rates of symptomatic reported infection between the sexes at different ages. MFM ratios were >1 for five viral infections out of seven in childhood, i.e. male children were more frequently reported as infected than females with pharyngoconjunctival fever, herpangina, hand-foot-and-mouth disease, mumps, and varicella. More males were also reported to be infected with erythema infectiosum and exanthema subitum, but only in children 1 year of age. By contrast, in adulthood the MFM ratios decreased to <1 for all of the viral infections above except varicella, i.e. adult women were more frequently reported to be infected than men. Sex- and age-related differences in reported morbidity were also documented for bacterial infections. Reported morbidity for enterohemorrhagic *Escherichia coli* infection was higher in adult females and females were reportedly more infected with mycoplasma pneumonia than males in all age groups up to 70 years.

**Conclusions:**

Sex-related differences in reported morbidity for viral and bacterial infections were documented among different age groups. Changes in MFM ratios with age may reflect differences between the sexes in underlying development processes, including those affecting the immune, endocrine, and reproductive systems, or differences in reporting rates.

## Introduction

Manifestation and morbidity of infections differ between the sexes and among different ages. For example, among viral infections, commonly accepted risk factors for enterovirus infections such as herpangina and hand-foot-and-mouth disease (HFMD) include age younger than 1 year and male sex, suggesting a predominance in male infants [1]. Further support for this idea came from a study on HFMD outbreaks in China [2], which demonstrated that boys were more susceptible than girls with the odds ratio of 1.56 (95% confidence interval [95%CI] 1.56–1.57). Although the major bulk of patients with erythema infectiosum (EI) are school-aged children, mothers are more infected than fathers, suggesting a female predominance in adulthood [1]. In addition, more patients with arthropathy, a major complication of EI in adults, are female than male [1], [3]. More girls than boys also acquired human herpesvirus (HHV-6), the pathogen of exanthema subitum (ES), in childhood; the acquisition of the virus was associated with female sex with the adjusted hazard ratio of 1.7 (95%CI 1.2–2.4) [4]. By contrast, more males contracted mumps in a large outbreak in Bosnia and Herzegovina in 2010–2011, a pattern which was also seen in the 2010 outbreak of rubella in Bosnia and Herzegovina [5].

With regards to bacterial infections, there was a reported male predominance in the incidence of tuberculosis across all age groups except for 15–24 year olds [6]. In contrast to that, the morbidity of pertussis is slightly higher in adult females [7], and during a recent German outbreak of *Escherichia coli* O104 in 2011, the majority of infected people were adults, and women were infected at about twice the rate as men [8], [9].

There are several studies describing age-specific sex-related differences in morbidity. The authors recently demonstrated that for influenza (H1N1) 2009 and seasonal influenza, the reported morbidity rate for males under twenty years old was statistically higher than that for females, while the relationship was reversed in adulthood [10]. Similar male predominance early in life and reversal at later ages were observed in human T-cell leukemia virus type I (HTLV-I) infection from blood donor data [11]. Thomas and Hall described age-specific sex-related differences in the morbidity of herpes zoster in the US [12], while Wu showed the annual incidence rates of chickenpox by age-group and sex, as well as the relative risks between sexes, using a large-scale database in Taiwan [13].

But studies which describe both age-specific and sex-related differences in morbidity are exceptional; the phenomenon is poorly documented in the literature as a recent review by the authors demonstrated. For example, one study reported that boys were more likely to be infected with adenovirus than girls, but no age-specific incidences were given by sex [14]. Reports by Zerr et al. [4] and Hukic et al. [5] described differences in morbidity for HHV-6 and mumps infections between the sexes, but did not mention age-specificity at all. In an analysis of serological surveys of the age-specific distribution of antibody to parvovirus B19, the pathogen of EI, no sex-related difference was described [15]. No sex-related age-specific difference in morbidity was mentioned in a survey on mumps in US [16]. Tan reported a thorough review on pertussis with international burden, but no description was given regarding sex-related difference in morbidity [17]. In a review on morbidity and mortality for vaccine-preventable diseases in the US, no description was given of sex-related differences in morbidity and mortality [18].

Even in a standard textbook of pediatrics [1] or handbook of communicable diseases [7], many infectious diseases are described with no information given about age-specific sex-related differences in their epidemiological profiles. Although peak ages of incidence or prevalence are detailed for many diseases, there is rarely any description of sex-based differences, including when such difference by sex occurs, until what age it continues, and what mechanism(s) might account for this effect. These shortcomings in knowledge can potentially hinder our understanding and control of infectious diseases.

The authors have developed a mathematical model based on nationwide data which enables the comparison of symptomatic reported morbidity rates of males and females by age groups [10]. The aim of the present study is to describe age-specific sex-related differences in infections using the Japanese nationwide infectious disease database and to consider sex- and age-related differences in viral and bacterial infections.

## Methods

### Ethics statement

Ethical approval and signed patient consent forms were not required for our study according to the Guideline for Epidemiological Studies [19], which was established by the Ministry of Health, Labor and Welfare and the Ministry of Education, Culture, Sports, Science and Technology of Japan in accordance with the World Medical Association's Declaration of Helsinki and Japan's Act on the Protection of Personal Information and other related acts. Specifically, (1) all individual data were collected by law and authorized to be utilized for academic purposes [20], and (2) patients could not be identified, as all data were de-identified; i.e., stripped of personal identifiers.

### Study population and data sources

Japan has an active infectious disease surveillance system. Since 1999, the National Institute of Infectious Disease (NIID; Tokyo, Japan) has collected reports of patients with various infectious diseases, and the data have been reported in sex and age groups (National Epidemiological Surveillance of Infectious Diseases, NESID) [21]. Diseases of interest in the present study were selected from those reported in NESID.

#### Viral infectious diseases without availability of vaccination reported from the pediatric sentinel points

Five major viral diseases are reported from the pediatric sentinel points of NESID: pharyngoconjunctival fever (PCF), herpangina, hand-foot-and-mouth disease (HFMD), EI, and ES. No vaccinations are available for these diseases in Japan. Data were collected from approximately 3000 pediatric sentinel points all over Japan between 2000 and 2009 (Table 1). The number of the sentinel points represents approximately 10% of the pediatric facilities in Japan, and the average number of sentinel points in 2009 was 3,022. As shown in Table 1, the numbers of male and female cases from the sentinel points were reported across 13 age groups, and adult cases were also reported.

**Table 1:** Numbers of cases and male-to-female morbidity ratios of viral infections reported from the pediatric sentinel points from 2000 to 2009 in Japan.

		Age	0	1	2	3	4	5	6	7	8	9	10–14	15–19	≥20
Infection without vaccination available	Pharyngo-conjunctival fever	Male	12767	42019	35164	39772	38487	30997	18169	10849	7224	4736	7718	674	2710
		Female	9828	31158	28769	32303	32039	25289	15768	9704	6495	4205	6290	623	6159
		M/F (95%CI)	1.23 (1.19–1.28)	1.28 (1.25–1.31	1.16 (1.14–1.19)	1.17 (1.15–1.20)	1.14 (1.11–1.16)	1.17 (1.14–1.20)	1.10 (1.06–1.13)	1.06 (1.02–1.11)	1.06 (1.01–1.11)	1.07 (1.01–1.14)	1.17 (1.11–1.23)	1.03 (0.88–1.21)	0.47 (0.44–0.50)
	herpangina	Male	59707	156797	124871	102487	78634	52619	26410	14268	8337	5118	7668	943	2378
		Female	50588	136847	116722	95002	74895	48590	25267	14051	8228	5196	6677	925	4751
		M/F (95%CI)	1.12 (1.10–1.14	1.09 (1.08–1.10)	1.02 (1.01–1.03)	1.03 (1.01–1.04)	1.00 (0.99–1.01)	1.03 (1.01–1.05)	0.99 (0.97–1.02)	0.97 (0.93–1.00)	0.96 (0.92–1.01)	0.94 (0.89–0.99)	1.09 (1.04–1.15)	0.97 (0.85–1.11)	0.54 (0.50–0.58)
	hand-foot-and-mouth disease	Male	36820	148707	132150	108946	87667	61360	30191	15216	9401	5635	8159	528	2025
		Female	30282	118713	109409	88077	72002	48905	25046	13271	8256	5076	7319	678	7496
		M/F (95%CI)	1.15 (1.13–1.18)	1.19 (1.18–1.20)	1.15 (1.14–1.16)	1.18 (1.16–1.19)	1.16 (1.14–1.18)	1.19 (1.17–1.22)	1.15 (1.12–1.18)	1.09 (1.05–1.13)	1.08 (1.04–1.13)	1.06 (0.999–1.11)	1.06 (1.01–1.11)	0.74 (0.63–0.88)	0.29 (0.27–0.31)
	erythema infectiosum	Male	8592	12927	14981	24210	33013	36941	29507	23196	16828	11233	14160	286	1152
		Female	8308	11159	13856	22502	32320	35788	30213	24800	18581	12220	13923	729	8287
		M/F (95%CI)	0.98 (0.94–1.03)	1.10 (1.06–1.14)	1.03 (0.99–1.06)	1.02 (0.998–1.05)	0.97 (0.95–0.995)	0.98 (0.96–1.004)	0.93 (0.91–0.95)	0.89 (0.87–0.91)	0.86 (0.84–0.89)	0.87 (0.84–0.91)	0.97 (0.94–1.002)	0.37 (0.30–0.46)	0.15 (0.14–0.16)
	exanthema subitum	Male	373434	179507	13653	1909	658	436	342	291	209	157	199	30	58
		Female	358470	164154	12857	1837	601	378	309	224	207	130	158	14	116
		M/F (95%CI)	0.99 (0.98–1.00)	1.04 (1.03–1.05)	1.01 (0.98–1.05)	0.99 (0.90–1.09)	1.04 (0.89–1.23)	1.10 (0.90–1.35)	1.05 (0.84–1.32)	1.24 (0.96–1.60)	0.96 (0.72–1.28)	1.15 (0.82–1.62)	1.20 (0.88–1.63)	2.04 (0.80–5.19	0.54 (0.34–0.85)
Infection with vaccination available	mumps	Male	4594	36580	69515	108637	137520	130210	92099	60145	37935	24507	40165	3353	8123
		Female	3293	27218	57281	91629	118793	109838	79821	53468	34277	21889	35995	3952	16112
		M/F (95%CI)	1.32 (1.24–1.42)	1.28 (1.25–1.31)	1.15 (1.14–1.17)	1.13 (1.11–1.14)	1.10 (1.09–1.12)	1.13 (1.12–1.14)	1.10 (1.08–1.11)	1.07 (1.05–1.09)	1.05 (1.03–1.08)	1.07 (1.04–1.09)	1.06 (1.04–1.08)	0.81 (0.75–0.86)	0.54 (0.52–0.56)
	varicella	Male	109643	241632	234577	225454	196204	131974	66813	32708	19237	11455	18494	2108	7036
		Female	104740	218016	216904	204682	179400	117473	61408	30851	18679	11308	17515	1918	7222
		M/F (95%CI)	0.99 (0.98–1.01)	1.05 (1.04–1.06)	1.03 (1.02–1.04)	1.05 (1.04–1.06)	1.04 (1.03–1.05)	1.07 (1.06–1.08)	1.03 (1.02–1.05)	1.01 (0.99–1.03)	0.98 (0.95–1.01)	0.96 (0.93–1.001)	1.00 (0.97–1.04)	1.05 (0.95–1.14)	1.05 (0.996–1.10)
Male-to-female population ratio (2000–2009)			1.053	1.052	1.051	1.50	1.50	1.50	1.051	1.051	1.051	1.051	1.051	1.052	1.042

#### Vaccine-preventable viral infectious diseases reported from the pediatric sentinel points

Two vaccine-preventable viral infectious diseases, mumps and varicella, were studied using reports from the pediatric sentinel points of NESID. Vaccinations for mumps and varicella are optional for children older than 1 year under the Japanese law with vaccination rates being 23.2% and 21.3% against mumps and varicella, respectively [22]. The vaccination rates for those two infections are only available for combined males and females - vaccination rates for males and females were not available separately. However, it is assumed that there are no differences in the vaccination rates between the sexes, based on data from vaccination rates for the measles-rubella combination vaccine in Japan where rates are available for each sex, and there is no difference in the vaccination rates between the sexes [10], [23].

#### Bacterial infectious diseases

Data for four bacterial infections were available from NESID for the present study (Table 2); Group A streptococcal pharyngitis (GAS), pertussis, enterohemorrhagic *Escherichia coli* (EHEC), and Mycoplasma pneumonia (MP). GAS and pertussis were reported from the pediatric sentinel points. EHEC cases must, by law, be reported by all clinical facilities in Japan and archived in NESID, while the data for MP were collected from approximately 470 NESID sentinel points. Of these, only pertussis is vaccine-preventable; the vaccine is generally provided four times between 3 months and 7·5 years as a component of the diphtheria, tetanus and pertussis combined vaccine which is recommended under Japanese law with a vaccination rate of 95.8% [22].

**Table 2:** Numbers of cases and male-to-female morbidity ratios of bacterial infections reported from the sentinel points from 2000 to 2009 in Japan.

	Age	0	1	2	3	4	5	6	7	8	9	10–14	15–19	≥20
group A streptococcal pharyngitis	MaleFemaleM/F (95%CI)	693258651.12 (1.07–1.18)	30523243001.19 (1.16–1.22)	57869460381.20 (1.17–1.22)	111793855651.24 (1.23–1.26)	1710591333861.22 (1.21–1.23)	191417	1567171475361.14 (1.13–1.16)	1138581011951.07 (1.06–1.08)	80190741561.03 (1.01–1.04)	54206515051.00 (0.98–1.02)	92088809501.08 (1.07–1.10)	730073610.94 (0.90–0.99)	11019479543600.45 (0.45–0.46)
pertussis	MaleFemaleM/F (95%CI)	409537371.04 (0.97–1.11)	150513841.03 (0.93–1.15)	6547500.83 (0.71–0.97)	6837330.89 (0.76–1.03)	5786830.81 (0.68–0.95)	5014681.02 (0.85–1.23)	3573990.85 (0.69–1.05)	3563470.98 (0.78–1.21)	3663610.96 (0.78–1.20)	3823431.06 (0.85–1.31)	123913800.85 (0.76–0.96)	2803760.71 (0.56–0.89)	13080154300.50 (0.46–0.54)
Male-to-female population ratio*		1.053	1.052	1.051	1.050	1.050	1.050	1.051	1.051	1.051	1.051	1.051	1.052	1.042

	Age	0	1–4	5–9	10–14	15–19	20–29	30–39	40–49	50–59	60–69	70≤
enterohemorrhagic *Escherichia coli*	MaleFemaleM/F (95%CI)	3392781.16 (0.92–1.46)	534443441.17 (1.10–1.24)	330926571.19 (1.10–1.28)	164512801.22 (1.10–1.36)	135512801.01 (0.90–1.13)	269830640.85 (0.78–0.91)	167222320.73 (0.67–0.81)	93712990.71 (0.63–0.81)	90416990.54 (0.48–0.61)	77811220.75 (0.65–0.86)	72813450.81 (0.71–0.92)
mycoplasma pneumonia	MaleFemaleM/F (95%CI)	6248360.71 (0.64–0.79)	12013132440.86 (0.84–0.88)	9691101700.91 (0.88–0.93)	435847820.87 (0.83–0.90)	7319680.72 (0.65–0.79)	78418420.41 (0.38–0.44)	72017600.40 (0.37–0.44)	3476490.53 (0.46–0.60)	2735250.53 (0.46–0.61)	3544510.85 (0.74–0.97)	8137551.60 (1.45–1.77)
Male-to-female population ratio		1.053	1.051	1.051	1.051	1.052	1.042	1.022	1.010	0.985	0.928	0.671

* identical to the bottom row of the Table 1.

### Statistical model and data analysis

#### Male-to-female morbidity ratios of infectious diseases without vaccine availability

Morbidities of males and females in each age group were compared through the male-to-female morbidity (MFM) ratios [10], statistics similar to ones used by Green [24] and Reller et al [25]. Since the present sampling is based on the data reported from the sentinel points, the sampling is viewed as a Poisson sampling. The morbidities (symptomatic incidence) at a current time, 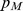 and 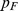, cannot be estimated from the observational patient data. Let 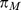 and 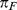 be the probabilities that male and female patients in the age group visit the sentinel points, respectively. From the present sampling from the sentinel points, the ratio 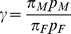 can be estimated by maximum likelihood estimator
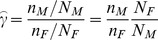
, where *N_M_* and *N_F_* are the subpopulations of males and females in an age group in Japanese population; i.e. fixed values, and *n_M_*, and *n_F_* are the random variables that describe the numbers of male and female patients. The ratio is referred to as the apparent MFM ratio. If 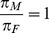, then, 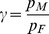 is the true MFM ratio. For large 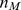 and 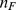, 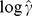 is asymptotically normally distributed with mean 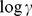 and variance 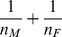. In order to make multiple tests of MFM ratios in age groups, the Bonferroni method [26] is employed, and the Bonferroni 95% joint confidence intervals of MFM ratios are constructed.

In order to estimate MFM ratios, ratios of male and female population sizes in age groups should be paid attention, as explained above. The male-to-female population ratios in age were almost constant from 2000 to 2009. Ratios of average subpopulations of males and females from 2000 to 2009 were used in this study (Table 1).

#### Male-to-female morbidity ratios of vaccine-preventable pediatric infectious diseases

Let 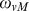 and 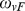 be the probabilities that male and female patients in the age group get vaccinated and let 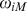 and 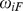 be the probabilities that vaccinated male and female patients in the age group get immunized, respectively. From the present sampling from sentinel points, the ratio 
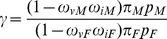
 can be estimated by maximum likelihood estimator
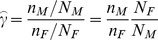
. If 
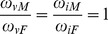
 and 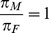, then, 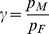 is the true MFM ratio.

## Results

### MFM ratios of viral infections

#### Viral infectious diseases without availability of vaccine

**Figure 1:**
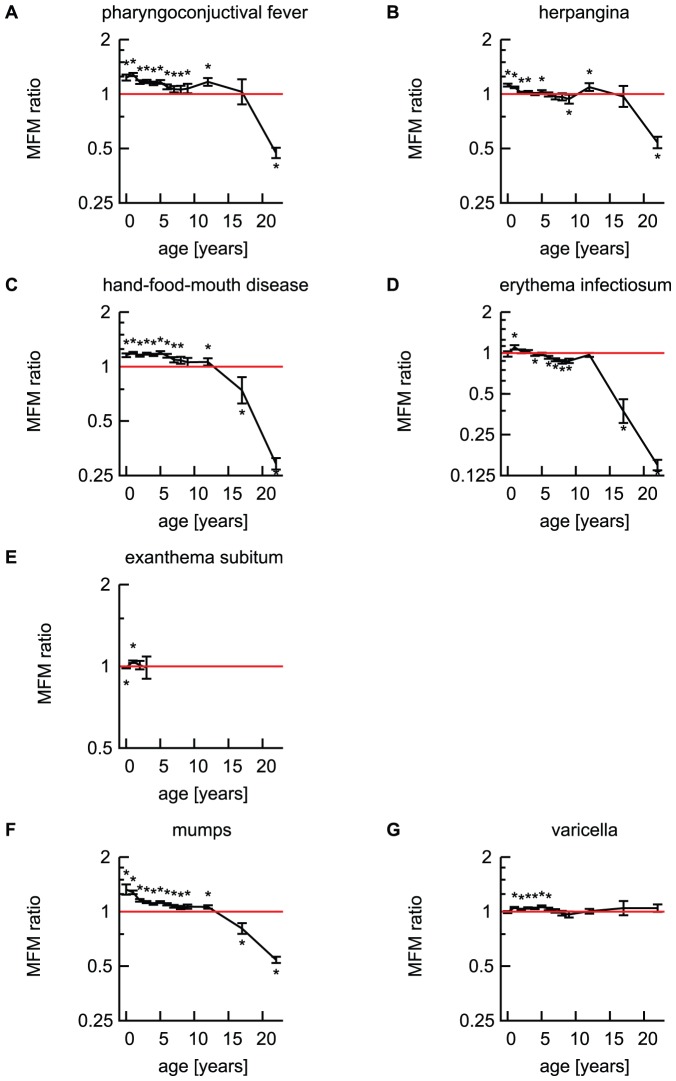
MFM ratios of viral infectious diseases reported from the pediatric sentinel points in Japan; pharyngoconjunctival fever (A), herpangina (B), hand-foot-mouth disease (C), erythema infectiosum (D) and exanthema subitum (E) for infections without availability of vaccination, and mumps (F) and varicella (G) for vaccine-preventable infections in Japan. 95% confidence intervals for MFM ratios are indicated by error bars. Red solid lines indicate MFM ratio of 1. *: Significant with the Bonferroni's correction (p<0·05/13) [26].

MFM ratios of five viral infectious diseases from NESID for which vaccination is not available are shown in Figure 1A–E. In this study, “children” refers to those aged younger than 15 years of age; “adolescence” 15–19 years of age and “adult” 20 years of age. In two diseases, MFM ratios of children under 15 years old were >1, i.e. male children under 15 years old were significantly more likely to be reported as infected with PCF, and HFMD (p<0.05, Figure 1A, C). The MFM ratios for reported cases of herpangina were >1 in 0–3, 5 and 10–14 years old (p<0.05, Figure 1B). The MFM ratios for reported EI was >1 only in children 1 year of age (MFM ratio 1.10, 95% confidence interval [95%CI] 1.06–1.14; Figure 1D). Of interest, MFM ratios for the above four diseases decreased to <1 by adulthood; i.e., by adolescence (15–19 years old), females were more frequently reported to be infected than males with HFMD (MFM ratio 0.74, 95%CI 0.63–0.88; Figure 1C), and by adulthood (≥20 year old) women were more affected than men by PCF (MFM ratio 0.47, 95%CI 0.44–0.50; Figure 1A) and herpangina (MFM ratio 0.54, 95%CI 0.50–0.58; Figure 1B). For EI, MFM ratios were <1 in 4, 6–9, and older than 15 years for age (Figure 1D). The MFM ratios for ES were 0.99 (95%CI 0.98–1.00) in 0 year and 1.04 (95%CI 1.03–1.05) in 1 year. In age groups over 4 years of age, the number of cases reported as “ES” were unreliable because ES is clinically unlikely in this group [1], [7]. Thus the data are not plotted in age groups ≥ 4 years of age in Figure 1E.

#### Vaccine-preventable viral infectious diseases

MFM ratios for reported cases of mumps were >1 from 0 to 14 years of age (p<0.05) and <1 thereafter (MFM ratio 0.82, 95%CI 0.76–0.87 in 15–19 years of age, Figure 1F). For reported cases of varicella, MFM ratios were >1 in 1–6 year old (p<0.05) and not different from 1 in newborns and those older than 7 years (Figure 1G).

### MFM ratios of bacterial infections

**Figure 2:**
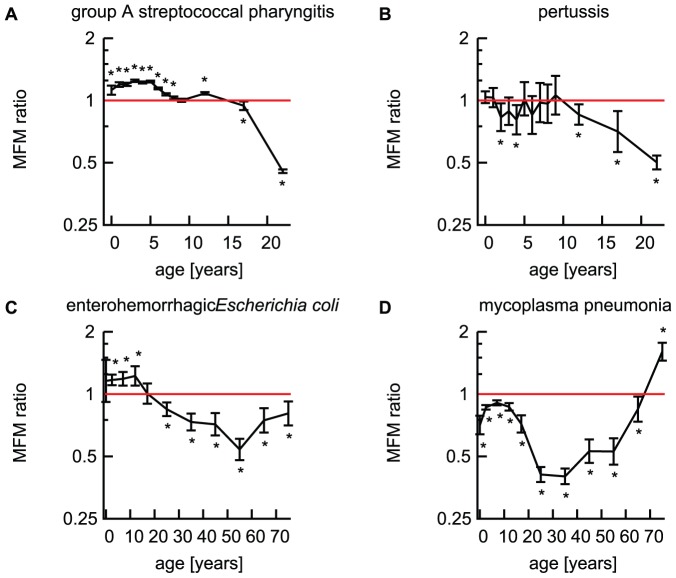
MFM ratios of bacterial infections; group A streptococcal pharyngitis (A), pertussis (B), enterohemorrhagic *Escherichia coli* (C) and mycoplasma pneumonia (D) reported from the sentinel points in Japan. 95% confidence intervals for MFM ratios are indicated by error bars. Horizontal red solid lines indicate MFM ratio of 1. *: Significant with the Bonferroni's correction (p<0.05/13 in A and B; p<0·05/11 in C and D) [26].

MFM ratios for reported cases of GAS were >1 in children under 15 years old (except 9 year olds) and <1 after adolescence (≥15 years of age, p<0.05); i.e. male children were reported significantly more often as infected than girls, but by adolescence, females were more frequently reported to be infected with GAS than males (MFM ratio 0.93, 95%CI 0.89–0.98 in 15–19 years of age; Figure 2A). In pertussis, the MFM ratios were <1 for children 2 years old, 4 years old, and over 10 years old (p<0.05, Figure 2B).

MFM ratios for EHEC were statistically >1 in 1–14 years old (p<0.05) and <1 in ages older than 15 years (MFM ratio 0.85, 95%CI 0.76–0.96 in 15–19 years of age; Figure 2C); i.e. boys under 14 years old were more frequently reported to be infected with EHEC than girls, whereas adult females were more reported to be infected with EHEC than males. The age category from 1 to 4 years old had the highest number of reported cases (Table 2).

As shown in Figure 2D, females are statistically more likely to be reported as infected with MP than males, except in elderly people above 70 years old. MFM ratios were the smallest in the age category from 30 to 39 years old (MFM ratio 0.40, 95%CI 0.37–0.44), while the highest number of reported cases is in those aged 1 to 4 years old (Table 2).

## Discussion

### Viral infections

The present study used the data of NESID, the national surveillance data of Japan, to demonstrate differences by sex and age in the symptomatic incidence of selected viral infections. The estimated incidence rates in Japan are as follows; PCF 22.2 per 1,000 population aged 0–14 years (95%CI 18.4–26.0), herpangina 51.7 (95%CI 47.8–55.6), HFMD 36.7 (95%CI 34.1–39.3), EI 15.2 (95%CI 13.9–16.6), ES 38.5 (95%CI 36.0–41.0), mumps 73.0 (95%CI 68.5–77.6), varicella 86.1 (95%CI 81.8–90.4) [27]. Statistical analysis of seven viral infectious diseases documented that male children are more likely to be reported as symptomatically infected than females in five out of the seven diseases (Figure 1A–C, F–G). Of these five diseases, the MFM ratios decrease to <1 by adulthood for PCF and herpangina, while those for HFMD and mumps were reversed to <1 in adolescence. For EI, the MFM ratio was 1.10 (95%CI 1.06–1.14) at 1 year of age, and ratios were <1 thereafter with smaller ratios ≥15 years of age. This might imply that there are age-specific sex-related differences in the immune response to viruses between childhood and adolescence/adulthood. However, every rule has its exception, and in this case the MFM ratio of varicella did not reverse to <1 in adults. MFM ratio for ES were <1 in infants and >1 at 1 year of age (p<0.05).

**Figure 3:**
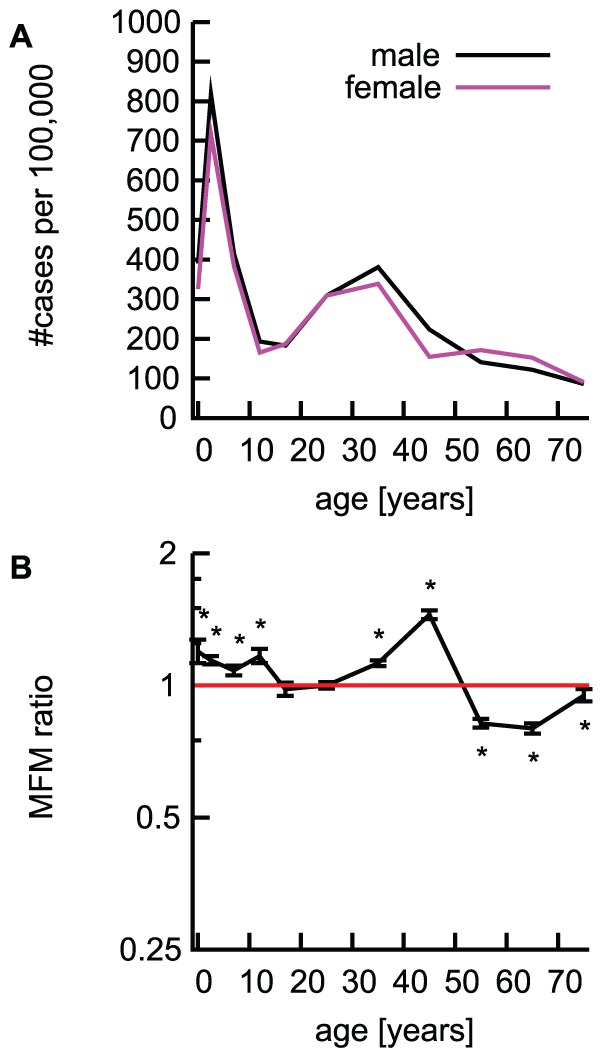
Epidemic keratoconjunctivitis (EKC) reported from the ophthalmological sentinel points in Japan. A: The number of reported cases of EKC (per 100,000 population) are illustrated for male (black) and female (red) separately. B: MFM ratios of EKC. 95% confidence intervals for MFM ratios are indicated by error bars, and horizontal red solid lines indicate MFM ratio of 1. *: Significant with the Bonferroni's correction (p<0·05/11) [26].

It is possible that the observed pattern is due to differences in social roles between the sexes; e.g. women may be more likely to take care of sick family members and thus are more likely to be affected by the disease [28]. Information about these diseases was obtained from ∼3,000 pediatric sentinel points; adult cases were also reported, probably because some accompanying parents might have consulted the pediatricians about their own health during their children's visit. Hence, there is a possibility that reported data for adults might have been gender-biased as it is more likely in Japan that mothers would be the accompanying parent. However, considering that MFM ratio was not different from 1 for varicella in adults (Figure 1G) and that the decreases of MFM ratios of some other diseases were observed from adolescence onwards (≥15 years of age; Figure 1C, D, and F), it is unlikely that the reversal of the MFM ratios was simply due to over-reporting by mothers from the pediatric sentinel points. Further evidence to the contrary comes from epidemic keratoconjunctivitis (EKC). The reported numbers of patients with EKC from the ophthalmological sentinel points of NESID (per 100,000) had two peaks at ages 1–4 and 30–39 years of age (Figure 3A), which may imply household transmission. However, MFM ratio was >1 (MFM ratio 1.12, 95%CI 1.11–1.14) in the age group 30–39 years old (Figure 3B), indicating that a bias due to accompanying parent gender would be unlikely.

### Bacterial infections

GAS with an estimated incidence of 66.6 per 1,000 (95%CI 60.5–72.6) [27] showed male predominance in childhood and the reversal in adolescence and adulthood. Morbidity of pertussis was never >1 in children or adults, indicating a female predominance at all ages as described elsewhere [7]. MFM ratios for EHEC were >1 in age groups 1–14 years of age and reversed to <1 in 15 year olds and older, i.e. boys were more likely to be reported as infected with EHEC than girls in childhood, while in adulthood, women were more likely to be reported as infected with EHEC than men. Total numbers of female and male cases with EHEC were 20,600 and 19,709, respectively, including 10,761 female and 7,717 male adult (≥20 years of age) cases (Table 2). Adults comprised 46% of the total cases, and 61% of the adult cases were in females. This is in accordance with the female adult preponderance in the 2011 outbreak of *Escherichia coli* O104 in Germany, where 87% of cases were adults and 68% of those adults were female [9].

*Mycoplasma pneumoniae* is the pathogen of MP with the second largest incidence rate of community-acquired pneumonia [29]. MP is unique among the bacterial infections examined in this study in that it showed a female preponderance in symptomatic infection at all ages except those over 70 years old (Figure 2D). In a population-based study on incidence of community-acquired pneumonia, it was reported that the incidence for *Mycoplasma pneumoniae* infection was not different in young and elderly people, and it was identical in males and females [29]. But the population size surveyed was ∼200,000 and it is possible that the test power was not sufficient.

### Strengths and weaknesses of the study

Several limitations of the present study should be noted. In general, observational studies do not verify the causality, because exposures to pathogens cannot be controlled. Covariates such as sex and age are confounded by human behavior, cultural influences, and other factors. The data used in the present study has a very large sample size collected through the official nationwide surveillance system in Japan [21]. Conservative 95% Bonferroni joint confidence intervals [26] of MFM ratios (Figures 1, 2 and 3) and assured precision of the estimates made it possible to demonstrate sex- and age-related differences in reported symptomatic infections.

It is possible that reporting rates are influenced by age and sex. In the model of the present analysis, the authors postulate that male and female patients consult physicians at the same rate. We further assume that parents seek health care equally for their sons and daughters. The latter assumption was based on the similar levels of immunization rates for boys and girls [23]. In 2008, male to female immunization ratios of measles-rubella combination vaccine were virtually 1 in children, indicating equality in vaccination rate [10], [23]. Identical medical care-seeking was reported in both sexes for salmonellosis in US [25]. Thus, we suggest a sex-based bias in the probability of seeking medical care during childhood is unlikely [10]. The authors believe that any bias in estimates of the MFM ratios introduced by age- and/or sex-based difference in reporting is minimal.

Accurate and precise estimate of morbidity rates depends on complete observation on the number of cases. Incomplete reporting of the number of infected individuals makes it difficult to accurately estimate morbidity rates [30]. The present study analyzed only data of symptomatic cases. The omission of asymptomatic cases might lead to biased results between males and females; it would also be possible that symptomatic to asymptomatic infection ratios differ by sex and age. In this study, the morbidities *P_M_* and *P_F_* are considered to be products of probability of transmission and probability of developing symptomatic disease, neither of which can be estimated separately.

The pathogens of the analyzed infections include both viruses and bacteria. Immunological responses against viruses and bacteria are different. It is therefore worth separately documenting sex- and age-related differences in the reported morbidity of viral and bacterial infectious diseases in order to understand differences in their respective immune responses.

### Putting research into context

This study documented examples of age-specific sex- related differences in morbidity for common infections. It also suggested a hypothesis that male children may be more susceptible to many of the common infectious diseases than female children, while this relationship is reversed by adulthood.

An apparent increased susceptibility of male children to selected infectious diseases has been frequently described [24]. But, with the exception of a limited number of studies [10]–[13], this increased susceptibility has been infrequently described in terms of sex and age as in the present study. In fact, some studies report no differences by sex for some diseases [15]–[18], for which significant differences were observed in this study. The merit of the methodology of the present study [10] is to show differences in morbidity by sex and age using observational data.

Genetic explanation for male-preponderance of infection in children has been proposed [31], [32]. As children grow, their body systems develop, including immune, endocrine and reproductive systems. Both innate and acquired immunity are influenced by reproductive hormones [33]–[39]. Changes in MFM ratios by age might reflect differences in the relative physiological development of immune, endocrine, and reproductive systems between male and female children as they grow.

Male-to-female differences in response to vaccination (including non-targeted effects) were reported from epidemiological cohort data [40]–[45], appreciating that the sex differences in immune responses could lead to more efficient vaccination programs [46], [47]. Despite data supporting an effect of sex in the response to vaccines, most studies do not document age-specific effects in vaccine efficacy or induced immune responses [47]. It is vital to understand sex- and age-related differences in the morbidity of infectious diseases in order to more efficiently prepare for and control outbreaks, investigate immune responses, and optimize disease-specific vaccine programs [47].

Population-based serological surveys studying antibodies (IgM and IgG) against pathogens or detecting pathogen DNA by polymerase chain reaction [4] would be more ideal in estimating accurate and precise infection rate. However, the cost of such investigations could pose a limiting factor in conducting a study using these methods. Another approach would be a large database where all cases of selected infections in a population are obligated to register, but the possibility of incomplete reporting (deliberate or unintentional) would still exist. The present study analyzed the data of NESID [21]. The sentinel points represent about 10% of all medical facilities in Japan, and the number of sentinel points in public health center areas are approximately proportional to their population size. Since reporting from the sentinel points is mandatory, the authors speculate that data from the sentinel points would proportionately represent the nation-wide trends.

In summary, this study provides evidence through the analysis of national data and calculation of MFM ratios that morbidity for viral and bacterial infections are sex- and age-dependent. Since our method uses observational data, we cannot avoid the possibility of under reporting which might confound the results [30]. However, under the proper circumstances, the methodology presented here may provide a powerful tool to study age-specific sex-related differences in the reported morbidities of selected diseases. These considerations have been poorly documented previously but have lately attracted more attention [48].
