# Correction: Age-Specific Sex-Related Differences in Infections: A Statistical Analysis of National Surveillance Data in Japan

**DOI:** 10.1371/annotation/e63213bd-8e30-4b2d-a37f-bcd1409f26ae

**Published:** 2012-10-09

**Authors:** Nobuoki Eshima, Osamu Tokumaru, Shohei Hara, Kira Bacal, Seigo Korematsu, Shigeru Karukaya, Kiyo Uruma, Nobuhiko Okabe, Toyojiro Matsuishi

The first two authors, Nobuoki Eshima and Osamu Tokumaru, should be noted as contributing equally to this work. 

